# Terbium-doped gadolinium garnet thin films grown by liquid phase epitaxy for scintillation detectors†

**DOI:** 10.1039/d5ra01784j

**Published:** 2025-06-04

**Authors:** Amandine Baillard, Paul-Antoine Douissard, Pavel Loiko, Thierry Martin, Eric Mathieu, Patrice Camy

**Affiliations:** a Centre de Recherche sur les Ions, les Matériaux et la Photonique (CIMAP), UMR 6252 CEA-CNRS-ENSICAEN, Université de Caen Normandie 6 Boulevard Maréchal Juin 14050 Caen Cedex 4 France pavel.loiko@ensicaen.fr +33 2 31 45 25 62; b European Synchrotron Radiation Facility (ESRF) 71 Avenue des Martyrs 38043 Grenoble France

## Abstract

Single-crystal films of terbium-doped gadolinium gallium garnet (Gd_3_Ga_5_O_12_:Tb) were grown by the isothermal dipping liquid phase epitaxy method on undoped (111)-oriented GGG substrates using PbO/B_2_O_3_ as a solvent. The effect of the Tb^3+^ doping level (2 to 10 at%) on the growth parameters, structure, composition, morphology, and emission properties of the films under optical and X-ray excitation was systematically studied. The saturation temperature increased almost linearly with the Tb content. The Tb^3+^-doped films exhibit a very low lattice mismatch of less than 0.05% with respect to the GGG substrate. The dopant ions are uniformly incorporated in the layers, with a segregation coefficient close to unity. The conversion efficiency of the films is optimized for a doping level of 6 at% Tb^3+^ in the solution, reaching a maximum light output of 52% with respect to a reference bulk YAG:Ce crystal. The green emission of Tb^3+^ ions at 543 nm matches with the maximum of sensitivity of CCD/CMOS sensors. The luminescence lifetime of the ^5^D_4_ Tb^3+^ emitting state amounts to ∼2.3 ms and is weakly dependent on the doping level. Minimum afterglow intensities are reached for the GGG:Tb films, as compared to other currently employed scintillators. Gd_3_Ga_5_O_12_:Tb single-crystalline films represent a viable solution for developing novel scintillators providing high efficiency and sub-μm spatial resolution for X-ray imaging.

## Abstract 2

Gd_3_Ga_5_O_12_:Tb single-crystalline films grown by liquid phase epitaxy represent a viable solution for scintillators providing high efficiency and sub-μm spatial resolution for X-ray imaging.

## Graphical abstract

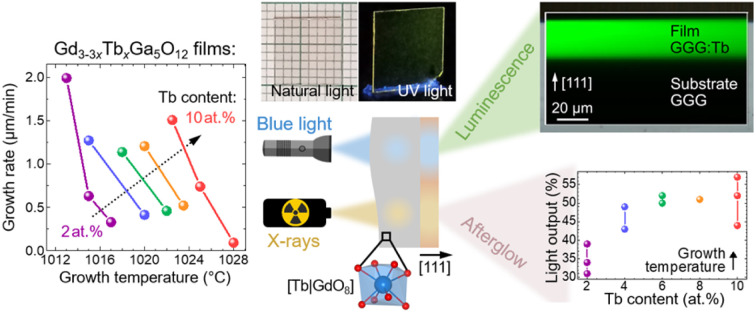

## 1. Introduction

X-ray detectors providing sub-μm spatial resolution consist of a single-crystal film (SCF) scintillator, light microscopy optics, and an image sensing device, such as a charge-coupled device (CCD) or a complementary metal–oxide–semiconductor (CMOS) sensor. The scintillator converts the incident X-rays into visible light that is then projected onto the sensor through the optics.^1^ X-ray detection is a non-destructive method, thus synchrotron-based techniques such as μ-tomography, holotomography or radiography find numerous applications in medical imaging and material engineering, *e.g.*, for the observation of phase contrasts, fatigue cracks, refractive index distribution, or corrosion.^2–4^

The spatial resolution *R* of such detectors can be adjusted through the ratio ∼0.61*λ*/NA, where *λ* is the emission wavelength of the detector, and NA is the numerical aperture of the optics.^1^ Moreover, high quality SCFs with a thickness of a few μm are required for X-ray imaging with sub-μm spatial resolution. Thicker films degrade the contrast for all spatial frequencies contained in the image.^5,6^

Nowadays, on top of high spatial resolution, detector systems offer the possibility to work at much higher speed with the use of faster CCD/CMOS sensors.^7^ The basic requirements for developing scintillators for high-resolution imaging have been detailed in the literature, with small variations depending on the targeted application. To combine both sub-μm spatial resolution and high frame rates (>50 frames per s), films with fast scintillation decay (<100 ns at the 1/*e* level) and especially low afterglow (0.01% after 2 ms) are necessary.^6,8^

Additionally, the ability to absorb X-rays is also a crucial parameter when selecting a host for a scintillator. This X-ray absorption capability is proportional to *ρZ*_eff_^4^, with *Z*_eff_ and *ρ* being the effective atomic number and the material density, respectively.^6^ Consequently, the absorption efficiency is improved by maximizing both parameters.^1^

Single-crystal film (SCF) scintillators have been elaborated at the European Synchrotron Radiation Facility (ESRF) for sub-μm spatial resolution X-ray imaging. The use of SCFs in X-ray imaging started with cerium-doped Y_3_Al_5_O_12_ (YAG:Ce) garnet films.^5^ They were soon replaced by Lu_3_Al_5_O_12_ (LuAG) and Gd_3_Ga_5_O_12_ (GGG) films doped with europium ions Eu^3+^, presenting higher densities and therefore higher efficiencies when integrated in X-ray imaging detectors.^6,9^ Starting in 2009, the novel Lu_2_SiO_5_ (LSO) SCF doped with terbium ions Tb^3+^ was developed at ESRF in the framework of the European project SCIN^TAX^,^10^ which provided better results in terms of spatial resolution and efficiency, especially in conjunction with back-illuminated sensors.^7,11,12^

The SCFs are elaborated using the Liquid Phase Epitaxy (LPE) method, which is well known for the growth of oriented epitaxial films and well adapted to garnets. It is a flux growth process in which the driving force of crystallization on a substrate is provided by the cooling of a supersaturated solution, consisting of a material to be grown (solute) in a suitable solvent. The growth temperatures are usually much lower than those for single crystals (SCs).^13–15^

The LPE method provides single-crystalline layers of very high optical quality with very few defects (inclusions, cracks). For photonic applications, it is often selected amongst other epitaxial growth techniques, *e.g.*, chemical vapour deposition or molecular beam epitaxy in vapour phase, as it provides nonporous layers at relatively high growth rates (in μm min^−1^).^16^ This growth technique presents numerous advantages, such as (i) the large available range of materials and dopants; (ii) the high available doping levels and uniform distribution of dopant ions; (iii) layer thicknesses from a few μm up to tens of μm can be reached on large surfaces of few cm^2^.

The gadolinium gallium garnet Gd_3_Ga_5_O_12_ (GGG) belongs to the crystal family of body-centred cubic multicomponent garnets with a chemical composition described by the general formula of {A}_3_[B]_2_(C)_3_O_12_, with {A}, [B], and (C) being the dodecahedral, octahedral, and tetrahedral symmetry cation sites. The GGG formula is represented as {Gd}_3_[Ga]_2_(Ga)_3_O_12_.^17,18^ Garnets are very flexible host matrices, with the possibility to extensively vary the chemical composition by substituting A, B and C cations to engineer the material properties. The {A} sites are appropriate for doping with lanthanide ions even with large ionic radii.^19,20^ Garnets are widely encountered in various applications, such as solid-state lighting and display technologies,^21^ solid-state lasers,^19^ scintillators for X-ray imaging,^6^ and electrolytes for Li-based batteries.^22^ The large density *ρ* of 7.1 g cm^−3^, as well as high effective atomic number *Z*_eff_ above 50 makes GGG an attractive candidate as host material for high quality thin-film scintillators.^1,6,8,23^

The SCFs presented in this study were doped with terbium ions. Trivalent terbium ions Tb^3+^ (electronic configuration: [Xe]4f^8^) provide multicolour emissions, from blue to deep-red, due to 4f–4f radiative transitions originating from the metastable state ^5^D_4_ to a set of lower-lying levels ^7^F_*J*_ (*J* = 6–0, with ^7^F_6_ being the ground state). The most intense emission of Tb^3+^ ions falls into the green spectral range around 543 nm, corresponding to the ^5^D_4_ → ^7^F_5_ transition. The large energy gap separating the ^5^D_4_ state from the next lower-lying level (about 15 000 cm^−1^) prevents depopulation of the emitting state by non-radiative multiphonon processes. Moreover, the ^5^D_4_ state presents a long luminescence lifetime, typically in the range of hundreds of μs to a few ms for garnets and more generally for oxide materials, depending on their crystal-field strength.^24–26^

Terbium-doped GGG was found to be an efficient green phosphor under both UV and X-ray excitation, thus being very interesting for solid-state lighting. This phosphor provided excellent quantum efficiencies comparable to commercially available ones, with yet a detrimental afterglow of several minutes under UV illumination. However, an afterglow of less than 1 s was observed under X-ray excitation.^23,27^

There exist studies on the X-ray imaging of the GGG:Tb SCFs produced by LPE.^28,29^ Jung *et al.*^28^ integrated a 6 μm-thick GGG:Tb scintillator in a fast μ-tomography system. This system allows to switch between high-resolution and high-speed acquisition mode. In the first one, the system achieved a breakthrough spatial resolution of 300 nm. In parallel, Douissard *et al.*^29^ studied the contrast and spatial resolution capabilities of a similar 6 μm-thick Tb^3+^-doped SCF in low-dose configuration. They obtained a contrast modulation in the order of 50% and a spatial resolution below 1 μm. Moreover, many synchrotron imaging beamlines use now routinely the GGG:Tb LPE scintillators for X-ray imaging applications.^30,31^

Nonetheless, no systematic study of the growth nor final properties of GGG:Tb SCFs was performed, unlike for other scintillator hosts such as YAG, LuAG or LSO.

In the present work, we report on the liquid phase epitaxy growth of Tb^3+^-doped GGG epitaxial layers for various doping levels, ranging from 2 to 10 at% Tb^3+^. We systematically studied the influence of the Tb^3+^ doping level on the growth parameters, morphology, composition, structure, and emission properties of the films under optical and X-ray excitation. The goal of this study is to further evidence the relevance of such films for developing novel scintillators providing sub-μm spatial resolution for X-ray imaging.

## 2. Experimental

### 2.1. Liquid phase epitaxial growth

Oriented layers of terbium-doped Gd_3_Ga_5_O_12_ (GGG) were grown at ESRF by the Liquid Phase Epitaxy (LPE) method on nominally pure (undoped) bulk GGG substrates using a mixture of lead oxide PbO and diboron trioxide B_2_O_3_ as a solvent. High-purity powders of Gd_2_O_3_, Ga_2_O_3_, Tb_4_O_7_, B_2_O_3_ and PbO were weighted, mixed, compressed in a cold isostatic press at 2000 bars, and finally placed into a platinum crucible (*Φ* 60 mm). The composition of the growth charge was carefully adjusted through the atomic ratios Pb/B and Ga/(Gd + Tb). The nominal doping level of Tb^3+^ ions with respect to Gd^3+^, *C*_Tb_ = Tb/(Tb + Gd), in the solution was progressively increased, from 2 to 10 at%, to determine the optimum doping level leading to the maximum luminescence efficiency of the SCFs. Moreover, an excess of gallium Ga was added to crystallize the garnet phase. The quasi-homoepitaxy of GGG:Tb films on undoped GGG substrates was performed without compensation of the lattice mismatch in order to stay close to the GGG density. The GGG substrates were elaborated *via* the Czochralski method.

The growth charge was first melted and homogenized at 1100 °C for several hours between each epitaxy. The molten bath was then stabilized at the growth temperature before dipping the substrate. The substrates were undoped GGG plates of 1 inch diameter with their plane orthogonal to the [111] crystallographic axis and a surface rugosity below 10 Å. The isothermal LPE dipping technique was employed in this study, *i.e.*, the substrates were alternatively rotated at 100 rpm into the solution in a horizontal position during the growth.^13^ Finally, the substrates were raised just above the solution and rotated at 800 rpm to remove any flux droplets.

The growth parameters are summarized in Table 1, for twelve growth attempts. The growth temperature was within the range of 1013 °C to 1028 °C, in the supersaturation domain of the solution. The growth duration was 10 min for epitaxies no. 1 to 10, 20 min for no. 11, and 4 min for no. 12.

**Table 1:** Growth parameters of the GGG:Tb layers by liquid phase epitaxy

Epitaxy no.	Duration (min)	Temperature^a^ (°C)	*C*_Tb_ b (at%)	*t* c (μm)	Growth rate (μm min^−1^)
1	10	1013	2	9.9	1.99
2	10	1015	2	6.3	0.63
3	10	1017	2	3.3	0.33
4	10	1015	4	12.7	1.27
5	10	1020	4	4.1	0.41
6	10	1018	6	11.3	1.13
7	10	1022	6	4.6	0.46
8	10	1020	8	12	1.20
9	10	1023.5	8	5.2	0.52
10	10	1025	10	7.4	0.74
11	20	1028	10	1.9	0.09
12	4	1022.5	10	6	1.50

a Growth temperature.

b Nominal doping level of Tb^3+^ ions in the solution, Tb/(Tb + Gd).

c Final thickness of the layer.

**Fig. 1:**
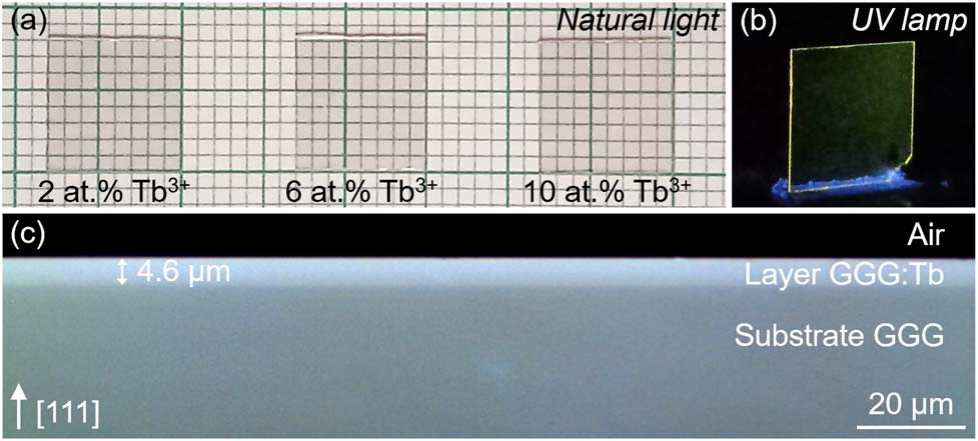
As grown GGG:Tb/GGG epitaxies: (a) photographs under natural light (epitaxies no. 3, 7, and 10, with 2 at%, 6 at%, and 10 at% Tb^3+^ in the flux, respectively); (b) a photograph under UV illumination, *λ*_exc_ = 313 nm (epitaxy no. 7); (c) a confocal laser microscopy image of an end-facet (epitaxy no. 7) using a ×50 objective.

Photographs of the as-grown epitaxies are shown in Fig. 1. They appear transparent under natural light, including the highly doped sample with 10 at% Tb^3+^, and the intense green emission from the dopant ions is visible under UV illumination. Additionally, a cross-sectional view of an epitaxy was examined using confocal laser microscopy to observe the clean and distinct interface between the substrate and the layer.

The final thickness of the SCFs was determined by weighting the samples before and after the growth. The layers thickness were not exceeding 15 μm during the LPE growth to limit the crystalline defects and especially the formation of cracks due to lattice mismatch tension. The density of the substrate and the layer were assumed to be similar and the growth uniform over the whole surface of the substrate. The growth on the substrate edges was neglected. This method provided a good agreement with the confocal laser microscope observations with a tolerance of ∼5%.

**Fig. 2:**
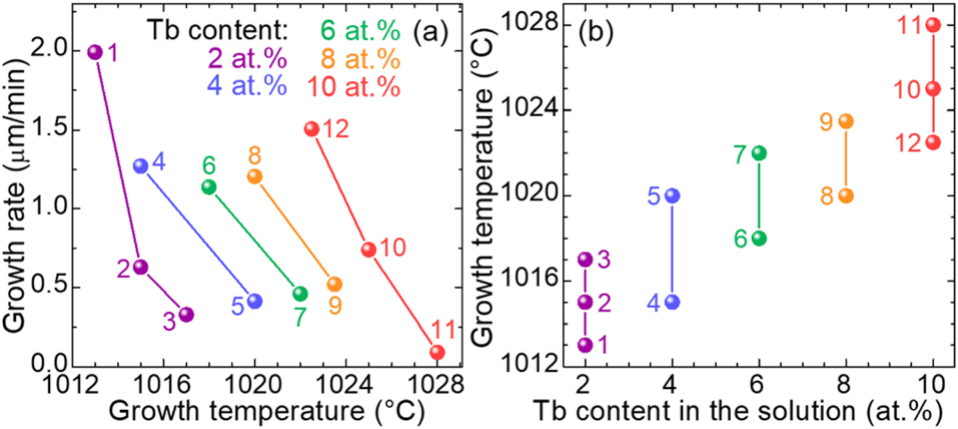
Influence of Tb doping level in the solution on the growth parameters of the GGG:Tb epitaxial layers: (a) variation of the growth rate with the growth temperature for various Tb doping levels; (b) range of the used growth temperatures for employed Tb doping levels. *Numbers* – epitaxy no., see Table 1.

The influence of Tb doping level in the flux on different growth parameters is presented in Fig. 2. For a growth duration of 10 min (epitaxies no. 1 to no. 9), and a given Tb content, the growth is slowed down on increasing the temperature (decreasing the supersaturation), see Fig. 2(a). For a growth duration above 15 min (epitaxy no. 11), the growth rate drops due to the depletion of solute in the vicinity of the substrate.

Moreover, the saturation temperature increases almost linearly with the Tb content, *i.e.*, between +2 °C and +3 °C every 2 at% of Tb^3+^, with the solute/(solute + solvent) ratio, see Fig. 2(b). The evaporation of the PbO/B_2_O_3_ solvent is also responsible for its increase.

### 2.2. Material

**Fig. 3:**
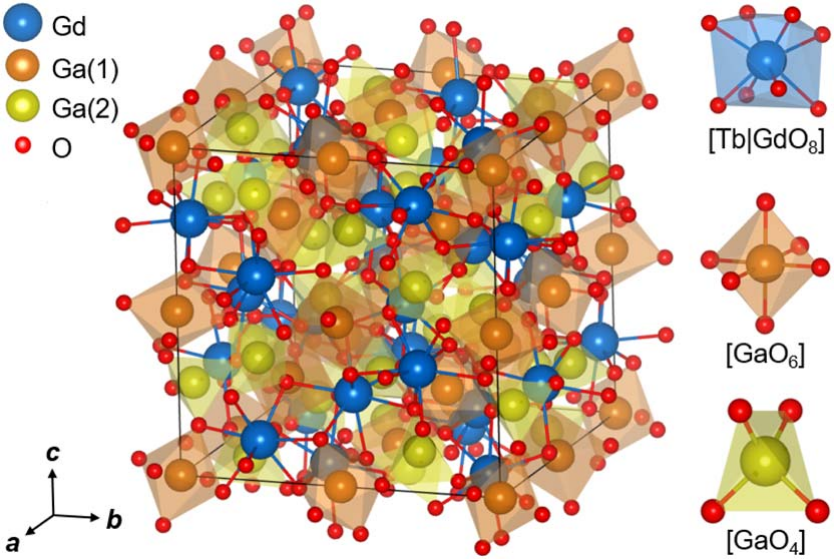
Crystalline structure of body-centered cubic Gd_3_Ga_5_O_12_:Tb (sp. gr. *Ia*3̄*d* – *O*_h_^10^) after the description reported in the literature:^33,34^: (*left*) a fragment of the structure within a single unit-cell (*black lines*); (*right*) the coordination polyhedra [Tb|GdO_8_], [GaO_6_]and [GaO_4_].

Undoped Gd_3_Ga_5_O_12_ crystallizes in the body-centered cubic space group *Ia*3̄*d* – *O*_h_^10^, similar to Y_3_Al_5_O_12_. The crystalline structure of GGG is depicted in Fig. 3. The trivalent gallium ions Ga^3+^ reside in two different types of sites. Within a single unit-cell, twelve Ga^3+^ ions are located in tetrahedral sites [GaO_4_] with *S*_4_ symmetry (Wyckoff position: 24d) and eight ions are found in octahedral sites [GaO_6_] (*C*_3i_ symmetry, 16a). There are twelve Gadolinium ions Gd^3+^ per unit cell, all residing in dodecahedral sites [GdO_8_] (*D*_2_ symmetry, 24c), being the substitutional sites for the rare-earth dopants such as Tb^3+^. Terbium ions Tb^3+^ being slightly smaller than Gd^3+^ ones (ionic radii: *r*_Gd_ = 1.053 Å and *r*_Tb_ = 1.040 Å for VIII-fold oxygen coordination^32^), the lattice parameter *a* of (Gd_1−*x*_Tb_*x*_)_3_Ga_5_O_12_ solid-solution compositions diminishes on increasing the Tb doping level, from 12.3772(2) Å for *x* = 0 (undoped) to 12.34121(2) Å for *x* = 1 (stoichiometric Tb_3_Ga_5_O_12_).^33^ Finally, the oxygen ions O^2−^ are the host-forming anions of the crystalline structure (Wyckoff: 96h).^17,18,33,34^

### 2.3. Methods

The X-ray diffraction (XRD) measurements were realized on the BM05 beamline at ESRF. The X-ray beam was monochromatized through a Si(111) monochromator (Δ*E*/*E* < 10^−4^). A two-axis diffractometer was used to record the (*ω*, 2*θ*) scans.

The composition of the layers was studied using an electron probe microanalysis (EPMA) setup equipped with five wavelength-dispersive X-ray spectrometers (WDS) and an analytical crystal of lithium fluoride LiF. The X-ray tube operated at 25 kV and 1000 nA. To measure the percentages of atoms constituting the SCFs, the epitaxies were coated with a resin and polished on one edge. The crystal was metalized with carbon to ensure the transport of charge through the sample surface, which is necessary for microprobe analysis. The measurements were performed by the French company “Bureau Veritas”. The percentages of atoms were measured with an accuracy of ±0.4%.

The μ-Raman and μ-photoluminescence spectra and the respective maps were recorded using a confocal Raman microscope (InVia Qontor, Renishaw) equipped with an Ar^+^ ion laser (488/514 nm) and a ×50 Leica objective. A 2400 l mm^−1^ diffraction grating coupled with a CCD matrix provided a spectral resolution down to 1 cm^−1^. The μ-Raman spectra were collected after 3 accumulations and 10 s integration.

The in-line transmission spectra were recorded with a spectrophotometer (Cary 5000, Agilent) providing a spectral bandwidth of 0.2 nm in the visible and 0.1 s integration.

The photoluminescence excitation spectrum, as well as the photoluminescence dynamics of Tb^3+^ ions were studied with a spectrofluorometer (QuantaMaster, Horiba). The excitation spectrum was recorded while monitoring the green luminescence from Tb^3+^ ions at 543 nm, using a spectral bandwidth of 0.4 nm in the UV-blue spectral range, and 0.8 nm in the visible, with 0.2 s integration. The photoluminescence decay curves were recorded while exciting Tb^3+^ ions at 488 nm and monitoring their emission at 543 nm.

The light output and the afterglow were recorded at room temperature with an X-ray generator using a copper anode. A 25 μm-thick copper filter was added to select the 8 keV Kα line of Cu. The X-ray tube delivered an X-ray photon flux density of 10^6^ photons per mm^2^ per s, over a dynamic range of 14 bit. The scintillator was mounted on a high-spatial resolution detector, consisting of microscope optics and a CCD camera. A dark-corrected flat-field image was employed to calculate the mean intensity in the image. The measurements were corrected for the X-ray absorption efficiency (accounting for the thickness of the scintillating layer), so the reported light output reflects the intrinsic conversion efficiency and is independent of the layer thickness. The CCD quantum efficiency was normalized with respect to a “bulk” YAG:Ce single crystal (light output: 30 000 ph per MeV, taking its conversion efficiency as 100%) because YAG:Ce scintillators have historically served as the reference for sub-μm spatial resolution imaging systems and are still widely used in X-ray beam monitoring applications at synchrotron sources. By “bulk,” we refer to a single crystal device with a thickness of 500 μm, which has been mechanically thinned down. Such scintillators provide an established performance baseline in terms of light yield and spatial resolution, making them a relevant reference for evaluating the performance of new thin-film scintillators. A comparison with SCF YAG:Ce would not be appropriate in our case, as its undoped YAG substrate also emits light under X-ray excitation. This parasitic luminescence interferes with our measurements and degrades the accuracy of the scintillator light output characterization. The afterglow signal decay was analysed with a photomultiplier (Philips XP2020Q) coupled to a SR400 gated photon counter working in counting mode, sampled at intervals of 8 ms using a SR445 amplifier (Stanford Research Instrument). The exposure time to X-ray was varied in the range of 0.1 s to 10 s, with a temporal resolution of 4 ms.

Measuring the light output of a thin-film scintillator using the pulse height spectrum method with X-rays (commonly used for bulk scintillators) is challenging due to: (i) low stopping power in thin-film scintillators at high energies (tens of keV), as most X-rays pass through without depositing energy, and without sufficient energy deposition, the scintillator does not emit enough light to measure a clear spectrum; (ii) poor energy resolution owing to a low light yield; (iii) poor light collection – most of the light is lost at the optical coupling, and the signal-to-noise ratio becomes too low for pulse height analysis; and (iv) electronic noise – the very low light yield from X-rays in the thin-film scintillator may be close to the noise level of the electronics (photodetectors). Tender X-rays in integration mode (the approach used here) are a better alternative for thin-film scintillators: this detection system is robust, reliable, and similar to the systems employed for X-ray imaging applications at synchrotron facilities. In addition, it provides information regarding imaging quality.

## 3. Results and discussion

### 3.1. Influence of Tb doping level on the crystal structure

**Fig. 4:**
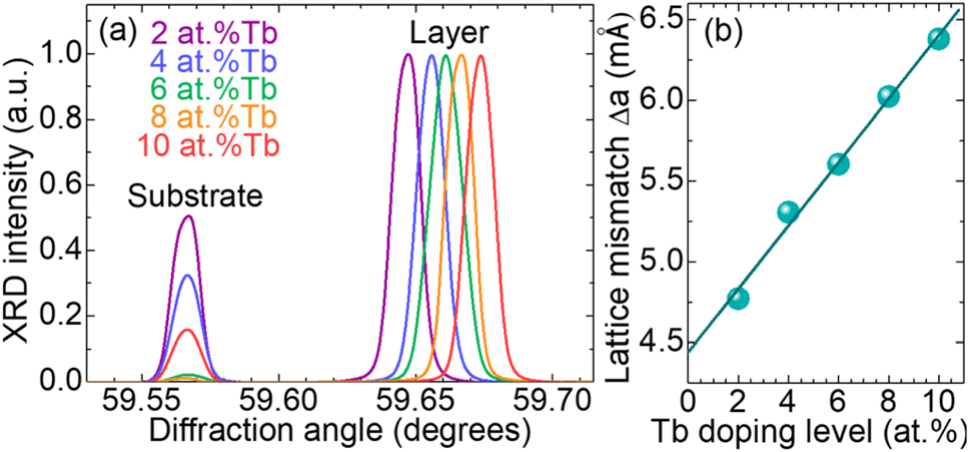
Influence of the Tb doping level on the lattice mismatch between the GGG substrates and the doped epitaxial layers: (a) X-ray diffraction (*ω*, 2*θ*) scans at the (888) reflection; (b) linear relation between the lattice mismatch and the Tb content.

As the doping level of terbium, *C*_Tb_, was progressively increased in the solution, the lattice mismatch between the epitaxial layer and the substrate was systematically studied through XRD measurements, as shown in Fig. 4(a). In order to distinguish the diffraction peaks of the layer and the substrate, the higher order reflection (*hkl*) = (888) was observed.

As mentioned above, Tb^3+^ ions are substituting Gd^3+^ ones in the dodecahedral sites of the GGG lattice, leading to a decrease of the lattice parameter *a* due to the difference of their ionic radii. In this case, the tension in the film with respect to the substrate increases with the content of Tb. The lattice mismatch, Δ*a*, between the doped epitaxial layer and the undoped substrate follows the Vegard's law:^35^ Δ*a* = Δ*a*_0_ + *α*_Tb_*C*_Tb_, where *α*_Tb_ = 0.20 is the hard-sphere diameter ratio of Tb^3+^, and Δ*a*_0_ = 4.44 mÅ the lattice mismatch between an undoped layer and the substrate.

For the same material, there is always a slight difference in the crystalline structure induced by the growth method. During the growth of the substrates by the Czochralski method, a small proportion of Gd^3+^ ions are found in the octahedral sites occupied in theory only by Ga^3+^ (the so-called antisite defects).^36^

Thus, the lattice parameter *a* of the GGG substrate is increased as compared to that of an undoped GGG layer grown by LPE, due to the larger size of Gd^3+^ ions (*r*_Gd_ = 0.938 Å and *r*_Ga_ = 0.620 Å in VI-fold oxygen coordination^32^). Therefore, Δ*a*_0_ is not zero in our case and there is a difference between the lattice of an undoped epitaxial layer and the GGG substrate.

As the Tb content is increased, the lattice mismatch follows the same trend, see Fig. 4(b). For the heavily-doped 10 at% Tb^3+^ SCFs, Δ*a* is reaching 6.38 mÅ, corresponding to a mismatch of Δ*a*/*a*_substrate_ = 0.05%, with the layers presenting higher densities of cracks. High quality garnet epitaxial films with very low surface roughness (down to a few nm) and low tensile stress can only be elaborated if the lattice mismatch with the substrate is very low (<0.1%).^14^

Furthermore, the diffraction peaks from the GGG:Tb layers are well defined and their full widths at half maximum (FWHM) are comparable to that of the substrate (approximately 0.012° and 0.011°, respectively), which reveals the excellent crystalline quality of the obtained layers.

### 3.2. Layer composition

**Fig. 5:**
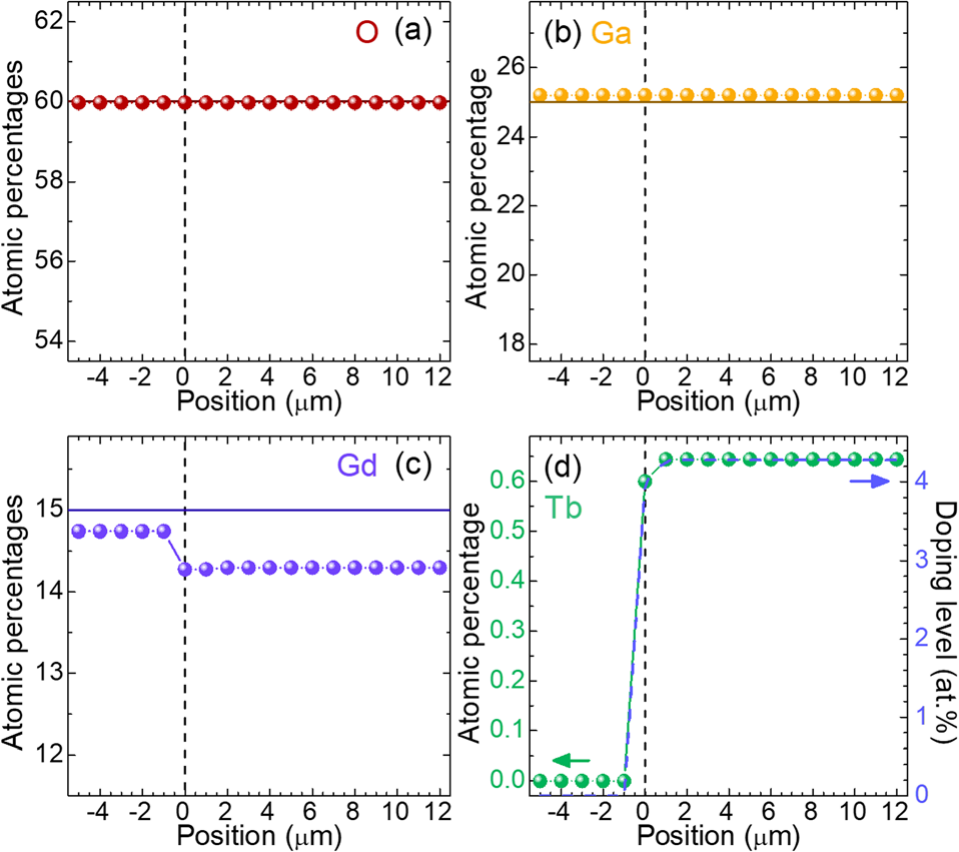
Percentages of atoms for the different constituents across the GGG:Tb epitaxy no. 4 with 4 at% Tb^3+^ in the flux: (a) O; (b) Ga; (c) Gd; (d) Tb. Positive positions correspond to the 12.7 μm-thick Tb^3+^-doped layer, and negative ones – to the GGG substrate. *Dashed vertical lines* – the layer/substrate interface. *Solid horizontal lines* – the stoichiometric composition for undoped GGG.

Electron probe microanalysis was conducted to study the composition of the GGG:Tb epitaxial films, by observing their edge. The atomic percentages of different constituents, namely O, Ga, Gd and Tb, are shown in Fig. 5. The studied SCF was grown from the starting composition with 4 at% Tb^3+^ and had a final thickness of 12.7 μm (epitaxy no. 4). The zero position corresponds to the interface where the layer started to grow, with positive positions being inside the layer and negative ones – inside the substrate. The composition of the layer is close to stoichiometry and the dopant Tb^3+^ ions are uniformly distributed through the entire thickness of the SCF. A slight decrease of the Gd^3+^ content evidences its substitution by Tb^3+^.

**Fig. 6:**
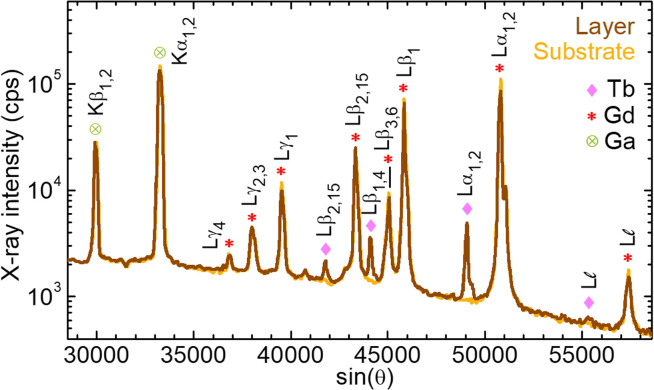
Comparison of the WDS spectra of a GGG substrate and a Tb^3+^-doped layer (epitaxy no. 4 with 4 at% Tb^3+^ in the flux). *Symbols* – contributions of Gd, Tb and Ga assigned using experimental data reported in the literature.^37,38^

The WDS spectra for the 4 at% Tb^3+^-doped GGG film and the GGG substrate are depicted in Fig. 6. The scatter peaks were assigned to the emission lines of Tb, Gd and Ga according to experimental results previously reported in the litterature.^37,38^ The incorporation of Tb^3+^ ions in the SCF is evidenced by the appearance of additional peaks assigned to Lℓ, Lα_2_, Lα_1_, Lβ_4_, Lβ_1_ and Lβ_2,15_ lines, corresponding to photon energies of 5.547 keV, 6.238 keV, 6.273 keV, 6.940 keV, 6.978 keV and 7.367 keV, respectively.^37^ The potential small contamination by Pb was below the detection limit.

The homogeneity of the layers was further studied by EPMA by measuring the atomic percentages at ten different positions over the surface (*Φ* 2.54 cm) of three SCFs doped with 4 at%, 6 at% and 10 at% Tb^3+^. The root mean square (r.m.s.) values are reported in Table 2. The content of dopant is uniform over the layer surface, as for the other constituents, as expressed by the small r.m.s. errors. The average actual Tb doping levels are 4.3 at%, 6.3 at% and 10.4 at% Tb^3+^. The segregation coefficient, *K*_Tb_ = *C*_layer_/*C*_solution_, is close to unity in all cases as expected due to the closeness of *r*_Gd_ and *r*_Tb_, highlighting excellent incorporation of the dopant ions in the GGG lattice, with only a slight reduction on increasing the doping level.

**Table 2:** Average values of percentages of atoms and segregation coefficients for Gd^3+^ and Tb^3+^ ions over the surface of the GGG:Tb epitaxies no. 4, 7 and 10, doped with 4 at%, 6 at% and 10 at% Tb^3+^, respectively

Epitaxy no.	*C*_Tb_ a	Gd	Tb	*C*_Tb,layer_ b	*K*_Tb_ c
4	4 at%	14.73 ± 0.81	0.66 ± 0.03	4.3 at%	1.07
7	6 at%	13.31 ± 0.17	0.90 ± 0.02	6.3 at%	1.05
10	10 at%	12.48 ± 0.22	1.45 ± 0.02	10.4 at%	1.04

a Nominal doping level of Tb^3+^ ions in the solution, Tb/(Tb + Gd).

b Actual doping level of Tb^3+^ ions in the layer.

c Segregation coefficient of Tb^3+^ ions, *C*_layer_/*C*_solution_.

The uniform distribution of the dopant both in the depth of the layer and over the surface ensures a very homogeneous response of the SCF under X-ray illumination.

### 3.3. Layer morphology

**Fig. 7:**
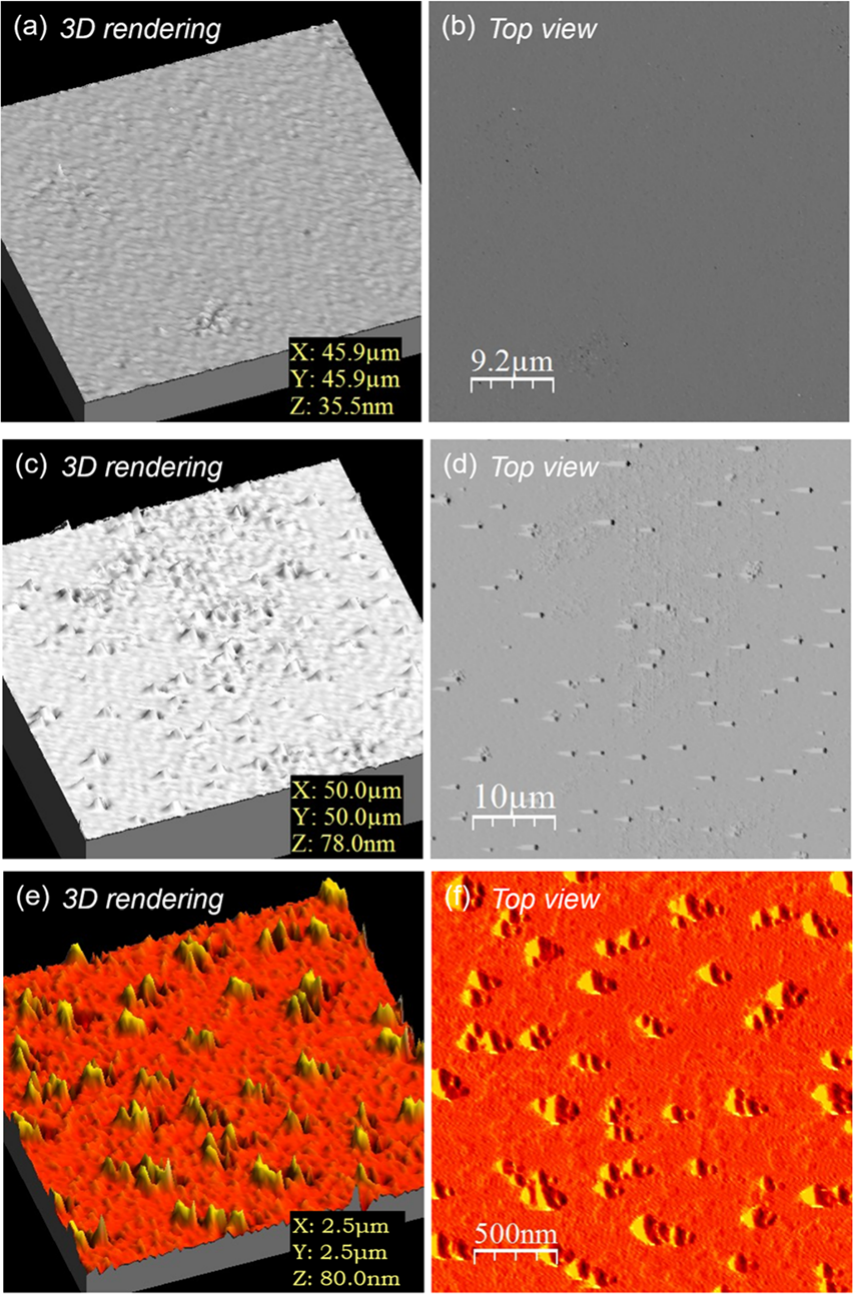
Atomic force microscopy images of the as-grown faces of a GGG:Tb layer (epitaxy no. 8): (a and b) the upper face over a 50 × 50 μm^2^ area: (a) 3D rendering, (b) top view; (c and d) the down face over a 50 × 50 μm^2^ area: (c) 3D rendering, (d) top view; (e and f) the down face over a 2.5 × 2.5 μm^2^ area: (e) 3D rendering, (f) top view.

The as-grown surfaces of the GGG:Tb SCFs were analysed by atomic force microscopy, and the obtained images are given in Fig. 7. As the isothermal LPE dipping technique was employed, with the samples rotated in a horizontal position, the two faces exhibit significantly different morphologies. In the zone free of flux droplets, the upper face of the layer appears very smooth, with a r.m.s. surface roughness of typically 0.4–0.5 nm over a 50 × 50 μm^2^ area, and peak-to-peak values of about 35 nm, see Fig. 7(a) and (b). On the contrary, the down face directed towards the solution presents a rougher surface with several pits and overgrowths, see Fig. 7(c)–(f). The r.m.s. roughness is increased to almost 4 nm, with peak-to-peak values of about 80 nm.

The down face is directed towards the solution and in contact with the PbO solvent vapours during the LPE growth, creating etch pits on the surface. This face is therefore removed by chemical-mechanical polishing and only the upper face is kept.

### 3.4. Raman spectroscopy

**Fig. 8:**
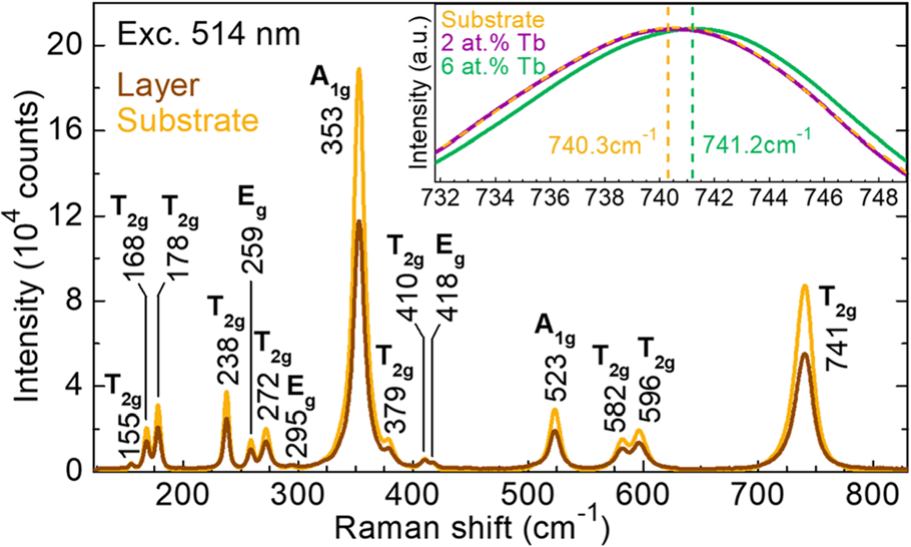
μ-Raman spectra of a GGG substrate and layer doped with 2 at% Tb^3+^ (epitaxy no. 3). The inset shows a close look at the Raman peak at 741 cm^−1^ for a GGG substrate and layers doped with 2 at% and 6 at% Tb^3+^ (epitaxies no. 3 and 6, respectively). *λ*_exc_ = 514 nm. *Numbers* – Raman frequencies in cm^−1^.

The Raman spectra of the Tb^3+^-doped GGG layer and the undoped GGG substrate are shown in Fig. 8, up to frequencies of 860 cm^−1^, corresponding to the range of one-phonon transitions. The factor group analysis predicts the following set of irreducible representations at the centre of the Brillouin zone *Γ*(*k* = 0) for cubic garnets: 18T_1u_ + 3A_1g_ + 8E_g_ + 14T_2g_ + 5A_2g_ + 5A_1u_ + 5A_2u_ + 10E_u_ + 14T_1g_ + 16T_2u_, with 25 even (*gerade*) modes being Raman active, namely A_1g_, E_g_, and T_2g_.^39^ The assignment of the Raman active modes was done according to the results reported by Mironova-Ulmane *et al.*^40^ for GGG SCs.

The high-energy range of the Raman spectra (*ν* > 500 cm^−1^) corresponds to internal vibrations of the [GaO_4_] and [GaO_6_] polyhedra, while the low-energy range (*ν* < 300 cm^−1^) corresponds to translation motions of the Gd^3+^ ions. The highest Raman frequency at 741 cm^−1^ is due to vibrations in antiphase of the [GaO_4_] and [GaO_6_] units, associated with a significant stretching of the bonds. Finally, the most intense peak at 353 cm^−1^ is due to separate rotations of these polyhedra around two different sets of perpendicular axes.^17,39,41^

The Raman spectra of the low-doped layer and the substrate are almost identical, except for a small decrease of intensity for the layer. The Raman peaks of the film also slightly broaden as compared to those of the substrate, with a FWHM of 17.9 cm^−1^ and 18.4 cm^−1^ for the substrate and the SCF at 741 cm^−1^, respectively. A close look at the highest Raman frequency mode located at 741 cm^−1^ is shown as an *inset* in Fig. 8 for a GGG substrate and layers doped with 2 at% and 6 at% Tb^3+^. A small blue shift of 0.9 cm^−1^ can be observed for the 6 at% Tb^3+^ doped layer with respect to the substrate. This phenomenon is explained by the variations of the crystalline structure by incorporation of dopant ions with a different ionic radius. The slight decrease of intensity, as well as the negligible broadening of the peaks for the layers confirm the excellent crystallinity of SCFs, with their crystalline structure almost identical to that of the substrate.

### 3.5. Optical spectroscopy

**Fig. 9:**
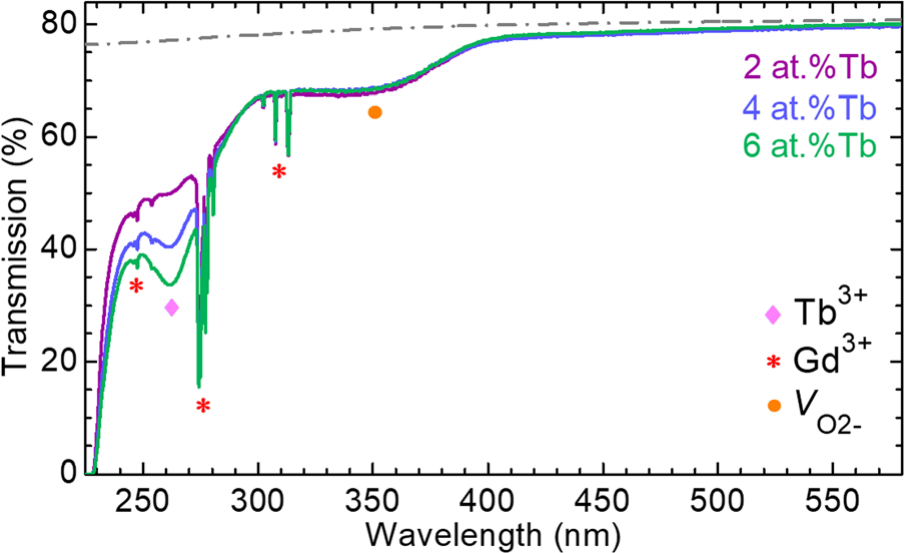
Transmission spectra of GGG:Tb epitaxies no. 3, 5 and 7 with 2 at%, 4 at%, and 6 at% Tb^3+^ in the flux, respectively, in the UV-visible spectral range. *Symbols* – contributions of Gd^3+^ and Tb^3+^ ions, and compensation of the oxygen vacancies V_O^2−^_, *dashed grey line* – theoretical limit *T*_0_ set by Fresnel losses after Wood and Nassau.^42^

The spectroscopic properties of dopant Tb^3+^ ions in GGG SCFs were studied, beginning with transmission measurements on the as-grown GGG:Tb epitaxies in the UV-visible range, Fig. 9. The epitaxies present high in-line transmittance in the emission range of Tb^3+^ ions (notably, in the green), measuring *T* = 79.6% at 545 nm. This value is close to the theoretical limit set by the Fresnel losses, *T*_0_ = 2*n*/(*n*^2^ + 1) = 80.7%, accounting for multiple light reflections at the sample interfaces, where *n* = 1.97 is the refractive index at 545 nm reported for undoped GGG by Wood and Nassau.^42^ The UV absorption edge is found at 230 nm (optical bandgap: *E*_g,opt_ = 5.39 eV). Undoped GGG presents a direct bandgap of 5.66 eV.^43^ The broad absorption band centered at ∼260 nm is attributed to inter-configurational parity allowed 4f^8^ → 4f^7^5d^1^ transitions of Tb^3+^ ions, as its intensity increases with the doping level.^44,45^

The presence of ionic impurities has been discussed in the previous works concerning garnet epitaxial films grown by LPE.^14,46–51^ In particular, Randoshkin *et al.*^48^ conducted a systematic study on the LPE growth of undoped GGG layers on (111)-oriented substrates of the same nature using a mixture of PbO/B_2_O_3_ as a solvent. They observed broad additional lines in the optical absorption spectra of the epitaxial films, with maxima at 280 nm, 325 nm, and 550 nm, associated with Pb^2+^ and Pb^4+^ ions. The presence and intensity of these lines depend on the supercooling, Δ*T*, of the solution, *i.e.*, the difference between the saturation temperature, *T*_s_, and the growth temperature, *T*_g_. The best quality films are generally grown from low supercooling. There is also a risk of Pt^4+^ impurities coming from the platinum crucible. These ionic impurities can strongly affect the optical properties.^50^ Therefore, the broad and weak absorption band between 250 nm and 300 nm could be assigned to a small amount of Pb^2+^ ions. No additional broad bands associated with Pb^4+^ ions were observed at the wavelengths reported by Randoshkin *et al.*^48^

Additionally, the narrow lines located at 300–315 nm, 273–282 nm, and 244–249 nm correspond to 4f–4f electronic transitions of the host-forming Gd^3+^ ions in the GGG lattice.^23,45,52,53^

Finally, the weak absorption band in the 315–395 nm range is connected to the charge compensation of oxygen vacancies.^46,53^ The parity and spin forbidden 4f–4f transitions of Tb^3+^ are not visible due to the small thickness of the layers.

**Fig. 10:**
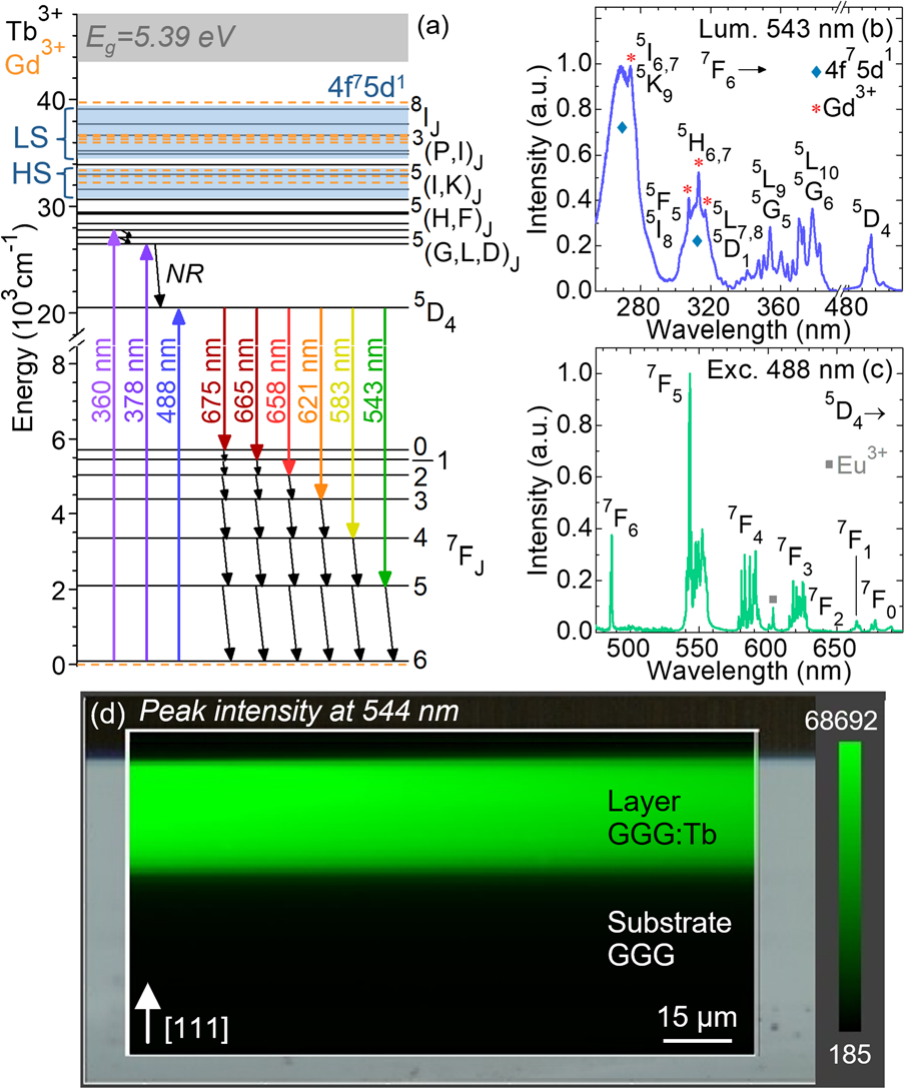
Excitation and luminescence properties of Tb^3+^ as dopant in GGG layers grown by LPE: (a) partial energy-level scheme of Tb^3+^ (*solid black lines*) and Gd^3+^ ions (*dashed orange lines*) after Carnall *et al.*,^54,55^*arrows* – 4f–4f transitions in absorption and emission of Tb^3+^, NR – multiphonon non-radiative relaxation, *grey area* – conduction band of Tb^3+^, *blue areas* – excited configuration 4f^7^5d^1^, LS and HS – low- and high-spin states, respectively; (b) photoluminescence excitation spectrum of GGG:Tb epitaxy, *λ*_lum_ = 543 nm, * – Gd^3+^ 4f–4f excitation lines, *diamonds* – Tb^3+^ inter-configurational 4f^8^ → 4f^7^5d^1^ transitions; (c) photoluminescence spectrum of Tb^3+^ ions, *λ*_exc_ = 488 nm, *square* – Eu^3+^ impurities in the GGG substrate; (d) μ-luminescence mapping across the end-facet of the GGG:Tb/GGG epitaxy monitoring the peak intensity at 544 nm (the ^5^D_4_ → ^7^F_5_ transition). The detailed (b) excitation and (c) luminescence spectra are available in the ESI materials.†

The scheme of both 4f and 5d energy levels of Tb^3+^, as well as 4f ones of Gd^3+^ ions are shown in Fig. 10(a) after Carnall *et al.*^54,55^ It depicts the 4f–4f transitions in absorption and emission from Tb^3+^ ions.

One way to excite a rare-earth-doped photoluminescent material is to target the broad and intense absorption bands of the 4f^*n*−1^5d^1^ excited configuration.^56^ Contrary to the 4f^*n*^ levels, the positions of the 4f^*n*−1^5d^1^ states strongly depend on the crystalline environment.^57,58^ The barycenter energies of the 4f^*n*−1^5d^1^ states are mainly affected by the nature of ligands in the host matrix (the nephelauxetic effect). Blasse and Bril^56^ have demonstrated that the ligand electronegativity and symmetry influence the amplitude of the 4f^*n*−1^5d^1^ level extension. For Tb^3+^, this excited configuration is located at relatively low energies as compared to other trivalent lanthanides due to the more stable half-full 4f^7^ shell.^56,58,59^

During the transition in excitation of a 4f^*n*^ electron to the excited configuration 4f^*n*−1^5d^1^, the f–d electron coupling for the ions in the second half of the lanthanide series 4f^*n*^ (*n* > 7) gives rise to 4f^*n*−1^5d^1^ high-spin (HS) and low-spin (LS) states. For Tb^3+^ ions, this transition results in two multiplets ^9^D_*J*_ and ^7^D_*J*_, exhibiting higher (HS) and lower (LS) spin multiplicity than the 4f^*n*^ ground-state ^7^F_6_, respectively. Consequently, the ^7^F_6_ → ^9^D_*J*_ transitions are spin-forbidden and the ^7^F_6_ → ^7^D_*J*_ ones are spin-allowed by the selection rules of spin multiplicity (Δ*S* = 0).^57,59^ Both LS and HS states of Tb^3+^ ions are shown in the energy-level scheme. Their energies were determined from the photoluminescence excitation spectrum with an accuracy of ±5 cm^−1^.

The photoluminescence excitation spectrum of Tb^3+^ ions as dopant in a GGG epitaxial layer is depicted in Fig. 10(b). The assignment of 4f–4f transitions was done following Carnall *et al.*^54^ The measurement was performed while monitoring the green luminescence at 543 nm.

The band falling in the blue spectral range with a maximum at 488.4 nm is due to a transition to the metastable state ^7^F_6_ → ^5^D_4_. The numerous overlapping bands in the UV-blue spectral range between 330 nm and 390 nm are ascribed to transitions to the higher-lying ^5^L_*J*_, ^5^G_*J*_, and ^5^D_*J*_ manifolds.

The inter-configurational 4f^8^ → 4f^7^5d^1^ transitions of Tb^3+^ ions are contributing to intense excitation bands in the UV, see Fig. 10(b). The band at 300–325 nm corresponds to the spin-forbidden ^7^F_6_ → ^9^D_*J*_ transitions to HS states, and the most intense one at 255–290 nm – to the spin-allowed ^7^F_6_ → ^7^D_*J*_ transitions to LS states. The latter one was also observed in the transmission spectra, Fig. 9. These bands are overlapping with low-intensity 4f–4f lines to higher-lying manifolds of Tb^3+^. The energy ranges of the HS and LS states of Tb^3+^ in GGG SCFs are given in Table 3. These experimental values are compared with those previously reported for other Tb^3+^-doped garnets. The HS–LS splitting corresponds to the energy gap between the onsets, *i.e.*, the lowest energies *E*_i_, of the HS and LS bands.

**Table 3:** Lowest *E*_i_ and highest *E*_f_ energies (in cm^−1^) of the 4f^*n*−1^5d^1^ high-spin (HS) and low-spin (LS) states of Tb^3+^ ions as dopant in miscellaneous crystalline garnets

Material	HS		LS		Splitting	Ref.
	*E*_i_	*E*_f_	*E*_i_	*E*_f_		
1% Tb:LuAG	30 120	32 787	34 722	40 000	4602	60
5% Tb:YAG	30 303	31 447	34 483	40 161	4180	56
5% Tb:YGG	31 056	34 247	34 722	41 152	3666	56
6% Tb:GGG	30 769	33 557	34 364	39 370	3595	*This work*

The more covalent the host matrix is, the larger the 4f^*n*−1^5d^1^ extension will be, thus reducing the interactions with the 4f^*n*^ electrons. This induces a smaller HS–LS splitting.^60^

Our experimental values are well in line with the ones for Tb^3+^-doped LuAG, YAG and YGG. Note the significant lowering of the HS–LS splitting when Al^3+^ ligands are replaced by Ga^3+^ ones. The electronegativity *χ* of Ga^3+^, *χ*_4_ = 1.755 and *χ*_6_ = 1.579 in IV- and VI-fold coordination, respectively, is superior to that for Al^3+^, *χ*_4_ = 1.691 and *χ*_6_ = 1.513.^61^ Therefore, Ga–O bonds are more covalent than Al–O ones, thus reducing the HS–LS splitting.

Furthermore, Gd^3+^ cations of the GGG host matrix exhibit 4f–4f excitation lines at 274 nm, 307 nm, 313 nm and 317 nm, Fig. 10(b). These lines are also observed in the transmission spectra, Fig. 9. They are overlapping with the 4f^7^5d^1^ excitation bands of Tb^3+^ ions, thus indicating an efficient energy transfer from the host matrix to the excited configuration of dopant ions, followed by a fast relaxation of the latter to the ^5^D_4_ metastable state.^23,45,52,53^

The photoluminescence (PL) spectrum of Tb^3+^ ions in a GGG layer is shown in Fig. 10(c). The characteristic emission lines of the Tb^3+^ ions are observed in the visible spectral range, with a maximum in the green at 543.4 nm corresponding to the ^5^D_4_ → ^7^F_5_ transition. Moreover, the weak background emission between 600 nm and 606 nm is due to Eu^3+^ contamination of the undoped GGG substrate. However, the content of these Eu^3+^ impurities is supposed to be low considering no additional absorption lines were observed in the 390–395 nm range, see Fig. 9.^44,52^

Additionally, μ-luminescence mapping was performed across the end-facet of a GGG:Tb/GGG epitaxy while monitoring variations of luminescence peak intensity at 544 nm, as shown in Fig. 10(d). The results indicate a uniform distribution of Tb^3+^ ions inside the SCF, with no diffusion into the GGG substrate.

**Fig. 11:**
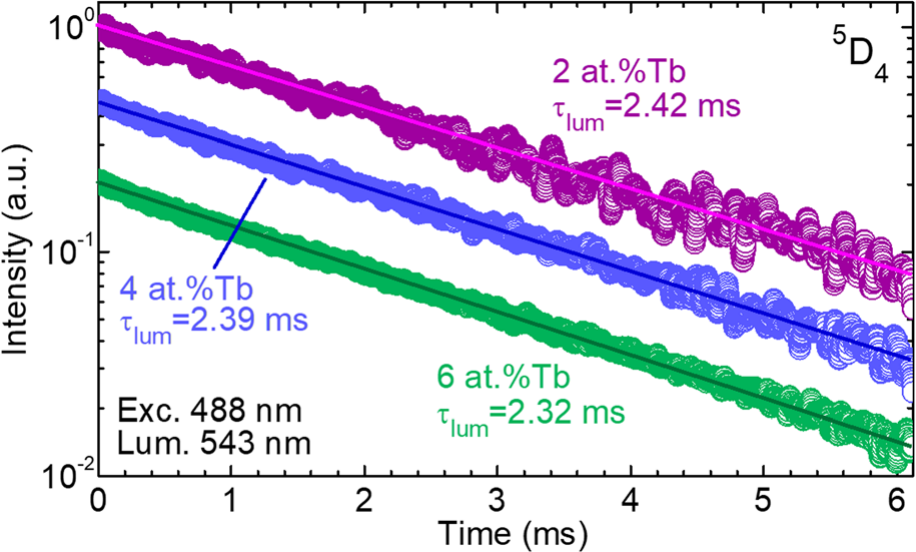
Photoluminescence decay curves from the ^5^D_4_ state of Tb^3+^ ions in GGG:Tb layers with various doping levels (epitaxies no. 3, 5 and 6, with 2 at%, 4 at%, and 6 at% Tb^3+^ in the flux, respectively). *λ*_exc_ = 488 nm, *λ*_lum_ = 543 nm. *Circles* – experimental data, *lines* – linear fit, *τ*_lum_ – luminescence lifetime of the ^5^D_4_ emitting state of Tb^3+^ ions.

The luminescence kinetics of Tb^3+^ ions were studied for various doping levels, as shown in Fig. 11. The luminescence lifetime of the ^5^D_4_ emitting state of Tb^3+^ ions was measured while exciting in the blue at 488 nm and monitoring the green emission at 543 nm. The ^5^D_4_ lifetime amounts to 2.42 ms for the low-doped (2 at% Tb) layer, being in line with those previously reported for Tb^3+^ in cubic garnets.^26,45^ It only slightly decreases down to 2.32 ms on increasing the doping level to 6 at% Tb^3+^, thus indicating the excellent crystalline quality of the layers with very few quenching centers. The luminescence lifetimes have been measured with an accuracy of ±0.05 ms. The decay curves are single exponential, in agreement with the single type of sites for Tb^3+^ ions in the GGG lattice (*D*_2_ symmetry).

### 3.6. Radioluminescence and afterglow

**Fig. 12:**
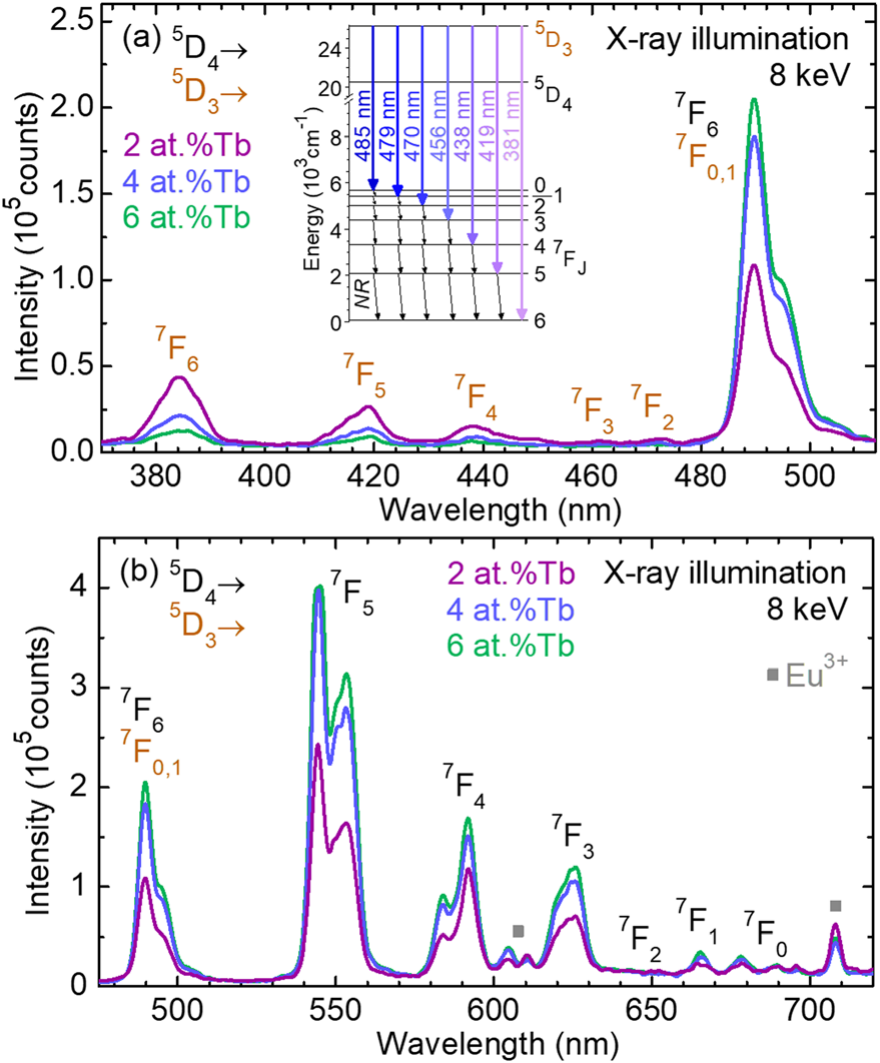
Radioluminescence spectra of Tb^3+^ ions in GGG epitaxial layers doped with 2 at%, 4 at%, and 6 at% Tb^3+^ in the flux, respectively: (a) UV-blue emission from the ^5^D_3_ manifold; (b) visible emission from the ^5^D_4_ manifold. X-ray illumination, 8 keV. *Squares* – Eu^3+^ impurities in the GGG substrate. The *inset* in (a) shows the partial energy-level scheme of Tb^3+^ after Carnall *et al.*,^54^*arrows* – 4f–4f transitions in emission from the ^5^D_3_ manifold, NR – multiphonon non-radiative relaxation.

Finally, the emission properties of the Tb^3+^-doped GGG epitaxial layers were studied under X-ray excitation. The radioluminescence spectra are displayed in Fig. 12, revealing emission lines characteristic of Tb^3+^ ions, originating from the ^5^D_3_ and ^5^D_4_ manifolds and terminating at the lower-lying ^7^F_*J*_ (*J* = 6–0) levels, falling in the UV-blue and visible spectral ranges, respectively. A partial energy-level scheme of Tb^3+^ is given as an *inset* in Fig. 12(a), deciphering emission lines from the ^5^D_3_ manifold. The ^5^D_3_ → ^7^F_0,1_ and ^5^D_4_ → ^7^F_6_ transitions are overlapping between 485 nm and 510 nm. On increasing the doping level, the blue emissions of Tb^3+^ ions becomes weaker, namely the ^5^D_3_ → ^7^F_2–6_ transitions, in favour of the ^5^D_4_ → ^7^F_*J*_ ones. This is most likely due to the strong cross-relaxation processes from the ^5^D_3_ state populating the metastable ^5^D_4_ one. Moreover, the absence of Gd^3+^ emission lines indicates an efficient energy transfer from Gd^3+^ ions of the host material to Tb^3+^.^45^

The substrate luminescence intensity is less than 1% of that for the layer under X-ray flux at 8 keV. However, the background luminescence of the substrate can become an issue on the synchrotron beamlines at high energies and high X-ray fluxes, as it can decrease the spatial resolution of the system.

As with the PL spectrum, weak parasitic lines associated with Eu^3+^ impurities in the GGG substrates were observed between 600–614 nm and 704–712 nm.

**Fig. 13:**
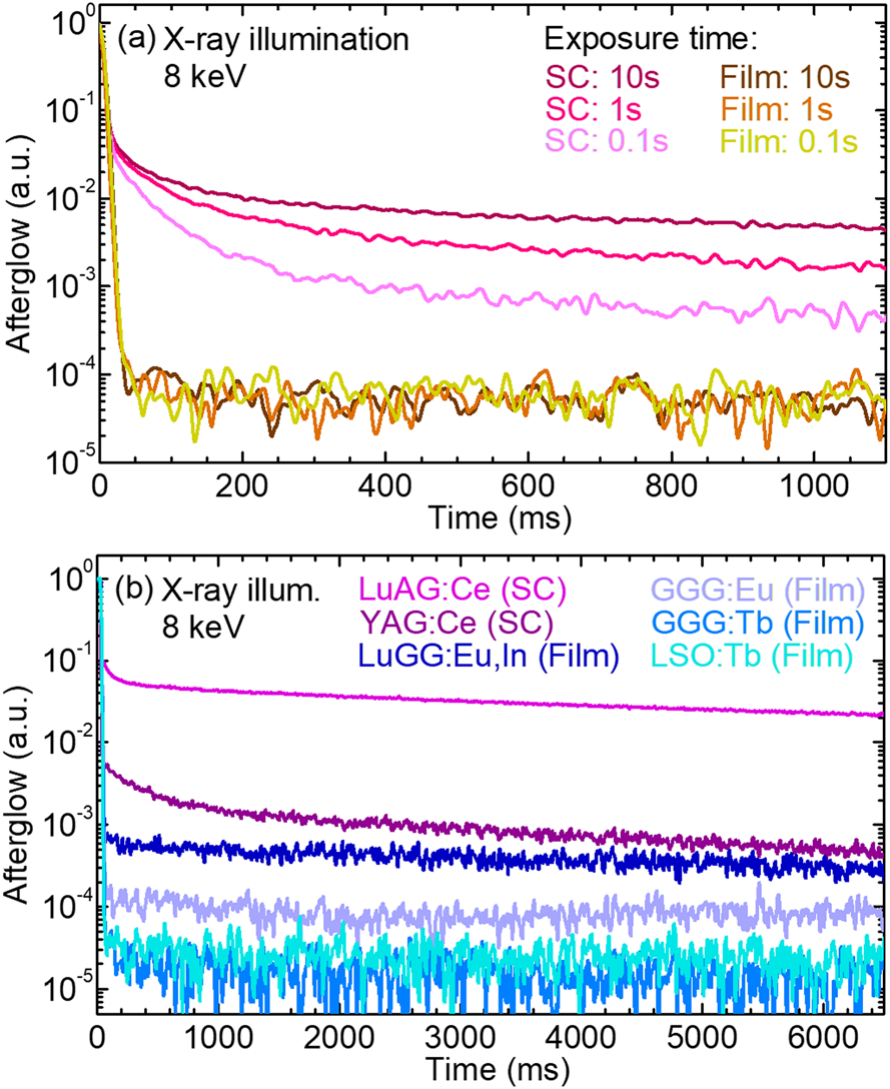
Afterglow properties of various rare-earth-doped garnet single crystals and single-crystalline films: (a) influence of the exposure time to X-rays on the afterglow of a GGG:Tb epitaxial film and a GGG:Tb single crystal, for exposure times of 0.1 s, 1 s and 10 s; (b) comparison of the afterglow for miscellaneous garnet single crystals and SCFs doped with various rare-earth ions, namely Ce^3+^, Eu^3+^ and Tb^3+^ (10 s exposure time). X-ray illumination, 8 keV.

Afterglow, the delayed luminescence from a scintillator after stopping its X-ray irradiation, is particularly detrimental for fast X-ray imaging applications.^11^ Since this phenomenon strongly depends on the exposure time to X-rays, its influence on the afterglow properties of GGG:Tb layers was studied. The results are given in Fig. 13(a), with exposure times of 0.1 s, 1 s and 10 s. The time response of the scintillators down to relative amplitudes of 10^−4^ to 10^−5^ was measured since a dynamic range up to 14 bit for successive images is required in some X-ray imaging applications. The afterglow of the GGG:Tb SCF was compared to that of its bulk SC counterpart which was grown by the Czochralski method. After 100 ms, the afterglow intensity of the SCF drops down to 6 × 10^−5^ in average, corresponding to a dynamic range of more than 14 bit. In the case of the GGG:Tb single crystal, the intensity decreases between 5.8 × 10^−3^ and 0.016 depending on the exposure time, which corresponds to less than 8 bit of dynamic range. The afterglow of the GGG:Tb layer is almost not detected in this explored dynamic range.

These results were compared with the performance of miscellaneous rare-earth-doped SCs and epitaxial layers based on garnets, see Fig. 13(b).

In general, the afterglow intensity for SCFs decreases faster and much further than for SCs, with a minimum value reached with the GGG:Tb layers studied in this work.

The Ce^3+^-doped LuAG and YAG SCs limit the dynamic range respectively to ∼4–5 bits and ∼7–8 bits after 100 ms. The higher afterglow in the case of SCs grown by the Czochralski method could originate from (i) impurities in the raw powders, and (ii) defects such as vacancies or antisites characteristic of this growth method at high temperatures.^36^

The afterglow of the Tb^3+^-doped films decreases much faster than that for other rare-earth-doped garnets already employed as scintillators.

**Fig. 14:**
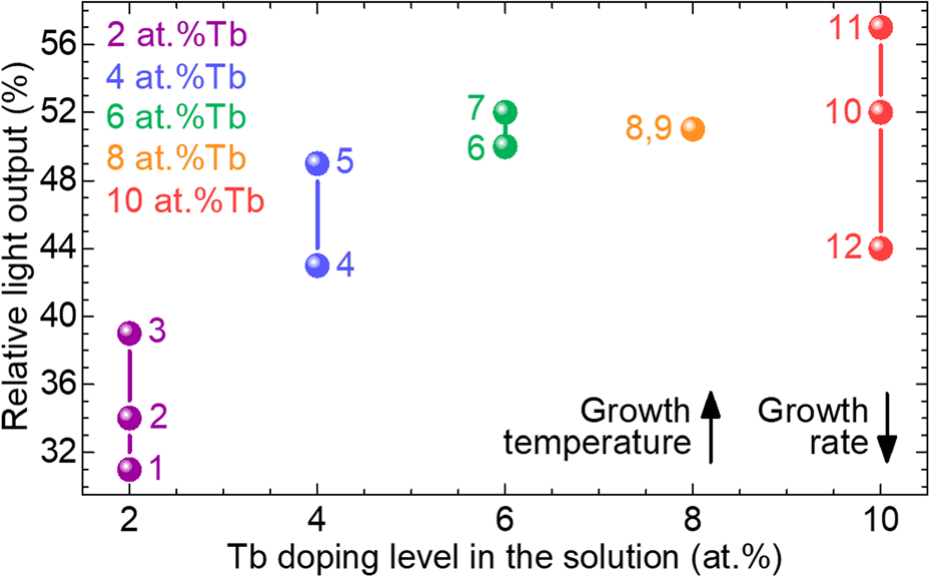
Influence of Tb doping level in the solution on the relative light output of the GGG:Tb epitaxial layers. *Arrows* – general evolution of the growth rate and temperature during the elaboration of the epitaxies. A 500 μm thick YAG:Ce single crystal scintillator was used as a reference.

An improvement of the light output was observed as the Tb doping level in the solution was increased, reaching a maximum of 52% at 6 at% Tb^3+^, see Fig. 14. The same optimal Tb content was determined by Lammers *et al.* for GGG:Tb powders.^23^ Above this doping level, the light output stabilizes at ∼51%. Note the exception of the epitaxy no. 11, doped with 10 at% Tb^3+^, reaching a maximum light output of 57%. This epitaxy was elaborated with the lowest growth rate of 0.09 μm min^−1^, thus limiting the incorporation of lead from the solvent. In fact, the light output is mainly influenced by the crystalline quality of the films, *i.e.*, by the growth parameters, as the presence of defects and impurities leads to non-radiative processes. Therefore, for a given Tb doping level, the light output slightly increases as the growth rate is lowered and the growth temperature is increased. This conversion efficiency corresponds to approximately 50% of that for a YAG:Ce single crystal.

## 4. Conclusions

The isothermal dipping liquid phase epitaxy method was employed to elaborate Tb^3+^-doped Gd_3_Ga_5_O_12_ single-crystal films on undoped (111)-oriented GGG substrates using PbO/B_2_O_3_ as a solvent. The effect of the Tb^3+^ doping level in the range of 2 to 10 at% on the growth parameters, structure, composition, morphology, and emission properties of the films under optical and X-ray excitation was systematically studied. The saturation temperature increased almost linearly with the Tb content in the solution. Monocrystalline films were obtained, exhibiting a good surface quality with a roughness of 0.5 nm under the optimal growth conditions. The GGG:Tb films are accommodated in tension on the GGG substrates, with a lattice mismatch of only 0.05% for 10 at% Tb^3+^ doping level, without the addition of buffer ions. The dopant ions are uniformly incorporated within the layers, with a segregation coefficient close to unity, and no diffusion into the substrate was observed, thus ensuring a homogenous response of the scintillators under X-ray illumination. A weak Eu^3+^ contamination of the GGG substrates was detected, as well as a small amount of lead impurities originating from the solvent. The latter can be minimized by lowering the growth rate.

The GGG:Tb epitaxies present high transmittance in the emission range of Tb^3+^ ions. The intense excitation bands corresponding to the inter-configurational 4f^8^ → 4f^7^5d^1^ transitions of Tb^3+^ ions are overlapping with low-intensity 4f–4f excitation lines in the UV spectral range. The experimental barycenter energies of the 4f^7^5d^1^ high-spin and low-spin states of Tb^3+^ in GGG films were determined by photoluminescence excitation measurements. Moreover, the 4f–4f excitation lines of the Gd^3+^ host-forming cations are also overlapping with the 4f^7^5d^1^ bands of Tb^3+^, revealing an efficient energy transfer from the host matrix to the dopant ions.

Terbium ions as dopant in the GGG layers exhibit their most intense emission in the green spectral range at 543 nm, fitting well with the sensitivity of CCD and CMOS sensors. The luminescence lifetime of the ^5^D_4_ Tb^3+^ emitting state amounted to 2.32 ms for 6 at% Tb^3+^, and is weakly dependent on the doping level. The conversion efficiency of the GGG:Tb films is optimized for a doping level of 6 at% Tb^3+^ in the solution, reaching a maximum light output of 52% with respect to a reference bulk YAG:Ce single crystal.

The Tb^3+^-doped layers do not exhibit any significant afterglow after X-ray irradiation at 8 keV, being absent from the films for a 15 bit dynamic range. Minimum afterglow intensities are reached for GGG:Tb films, as compared to other SCFs currently employed as scintillators. The luminescence intensity of the GGG substrates under X-ray illumination is very low, namely less than 1% of the Tb^3+^-doped layers.

Following these results, we suggest that the GGG:Tb epitaxial layers are attractive candidates for applications in X-ray imaging with sub-μm spatial resolution. Indeed, the films meet most of the criteria for high-resolution imaging scintillators, namely their excellent optical quality and high density, high transmittance in the spectral range of Tb^3+^ green emission, and the emission wavelength matching the spectral maximum of sensitivity of CCD/CMOS sensors. The afterglow can be further optimized by eliminating the impurities and crystalline defects introduced during the synthesis of the films.

## Data availability

The authors confirm that the data supporting the findings of this study are available within the article and its ESI materials.†

## Author contributions

P.-A. D. and E. M. fabricated the epitaxial films and performed their structural and compositional characterizations and the associated data analysis. A. B. and P. L. performed all the spectroscopic studies of the films. T. M. and P. C. conceptualized and supervised the project. The manuscript was written through contributions of all authors. All authors have given approval to the final version of the manuscript.

## Conflicts of interest

There are no conflicts of interest to declare.

## Supplementary Material

RA-015-D5RA01784J-s001
