# Supplementary material for: Terbium-doped gadolinium garnet thin films grown by liquid phase epitaxy for scintillation detectors
Source: RSC Adv. 2025 Jun 4;15(24):18802–13. doi: 10.1039/d5ra01784j (PMC12135170; doi:10.1039/d5ra01784j)
Supplement: RA-015-D5RA01784J-s001 [file RA-015-D5RA01784J-s001.pdf]

Supplementary Information (SI) for RSC Advances.
This journal is © The Royal Society of Chemistry 2025

Electronic supplementary information (ESI) for:

Terbium-doped gadolinium garnet thin films grown by Liquid Phase Epitaxy for scintillation detectors

Amandine Baillard,[‡a] Paul-Antoine Douissard,[‡b] Pavel Loiko,[*a] Thierry Martin,[b] Eric Mathieu,[b] and Patrice Camy [a]

[a] *Centre de Recherche sur les Ions, les Matériaux et la Photonique (CIMAP), UMR 6252 CEA-CNRS-ENSICAEN, Université de Caen Normandie, 6 Boulevard Maréchal Juin, 14050 Caen Cedex 4, France.*

* E-mail: pavel.loiko@ensicaen.fr

[b] *European Synchrotron Radiation Facility (ESRF), 71 Avenue des Martyrs, 38043 Grenoble, France.*

‡ Both are the first authors.

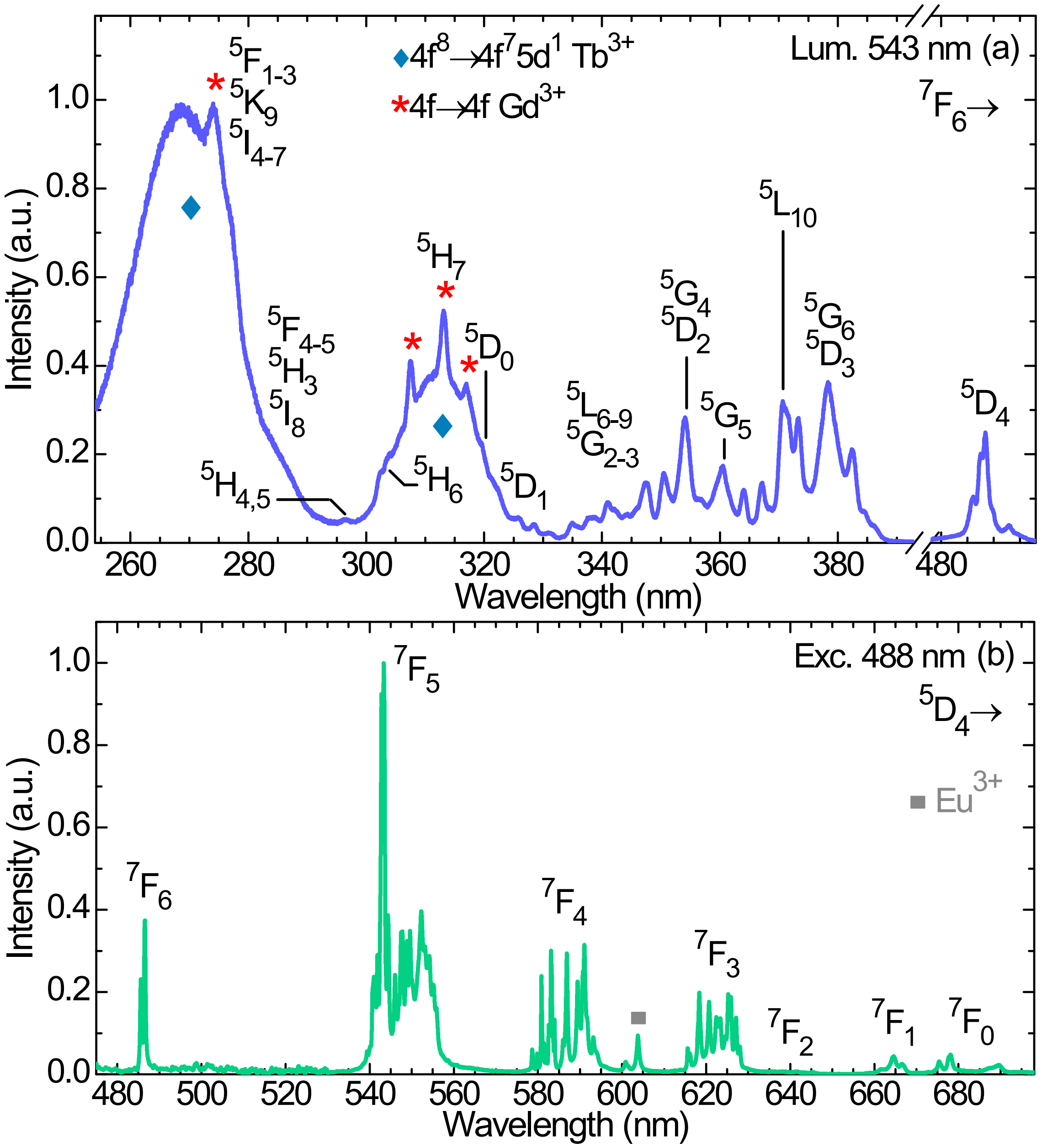

Fig. S1 Detailed excitation and luminescence properties of Tb^{3+} as dopant in GGG layers grown by LPE: (a) photoluminescence excitation spectrum of GGG:Tb epitaxy, λ_{lum} = 543 nm, * – Gd^{3+} 4*f*-4*f* excitation lines, *diamonds* – Tb^{3+} inter-configurational $4f^8 \rightarrow 4f^75d^1$ transitions; (b) photoluminescence spectrum of Tb^{3+} ions, λ_{exc} = 488 nm, *square* – Eu^{3+} impurities in the GGG substrate. The assignment is after Carnall *et al.*[1,2]

1 W. Carnall, P. Fields and K. Rajnak, *J. Chem. Phys., 1968,* **49**, 4447–4449.
2 W. Carnall, P. Fields and K. Rajnak, *J. Chem. Phys., 1968,* **49**, 4443–4446.
